# A Proposed Diagnostic Algorithm for Helicobacter pylori Infection in Cameroon: A Comparison of Five Diagnostic Methods in Patients With Gastroduodenal Symptoms

**DOI:** 10.7759/cureus.112689

**Published:** 2026-07-15

**Authors:** Stanley Ngemeshe Ngimgoh, Kobada Maaga Marina, Justine Laure Nguieguia Konang, Carole Marlyse Menzy Moungo-Ndjole, Jean Paul Dzoyem, Kouitcheu Mabeku Laure Brigitte

**Affiliations:** 1 Department of Biochemistry, University of Dschang, Dschang, CMR; 2 Department of Microbiology, University of Yaoundé I, Yaoundé, CMR; 3 Department of Endoscopy, Bafoussam Regional Hospital, Bafoussam, CMR; 4 Department of Anatomopathology, Yaoundé General Hospital, Yaoundé, CMR

**Keywords:** cameroon, diagnostic algorithm, diagnostic methods, helicobacter pylori infection, pcr

## Abstract

Introduction

*Helicobacter pylori* (*H. pylori*) infection remains an important cause of gastrointestinal disease worldwide. Both direct (polymerase chain reaction (PCR), histology, and rapid urease test (RUT)) and indirect (stool antigen test (SAT) and serology) diagnostic methods have been developed, yet each technique has its own limitations and merits. In Cameroon, there is no consensus guideline for *H. pylori* diagnosis, and diagnostic practices vary widely across healthcare settings. Consequently, establishing an accurate, context-appropriate diagnostic algorithm is imperative to facilitate effective management in our milieu. The present study aimed to compare the diagnostic methods for *H. pylori* currently used in Cameroon so as to propose a context-appropriate diagnostic algorithm for this infection.

Materials and methods

This cross-sectional, prospective, hospital-based study enrolled 82 dyspeptic adult patients presenting with gastroduodenal symptoms. Gastric samples were tested for *H. pylori* using five diagnostic techniques: PCR, SAT, histology, serology, and RUT. PCR was designated as the reference test for comparative analysis. Diagnostic performance of all tests was evaluated by calculating sensitivity, specificity, positive predictive value (PPV), negative predictive value (NPV), and accuracy, and discriminatory power between tests was evaluated using the area under the curve (AUC) with corresponding receiver operating characteristic (ROC) curves. Statistical analyses were performed using RStudio version 4.4.1 (RStudio, Inc., Boston, MA, USA).

Results

*H. pylori* infection detection rates were as follows: PCR, 79.26%; SAT, 62.2%; histology, 45.1%; serology, 76.8%; and RUT, 73.2%. Using PCR as the reference standard, the performance characteristics of sensitivity, specificity, PPV, and NPV of histology, RUT, SAT, and serology were 52.31%, 82.35%, 91.89%, and 31.11%; 76.92%, 41.18%, 83.33%, and 31.81%; 67.69%, 58.82%, 86.27%, and 32.26%; and 81.54%, 41.18%, 84.13%, and 36.84%, respectively. The accuracy of histology, RUT, SAT, and serology was 58.54%, 57.32%, 65.85%, and 73.17%, respectively. Discriminatory ability was significantly higher for histology (AUC: 0.673) compared to RUT (AUC: 0.405) (p < 0.001). SAT showed comparable discriminatory ability (AUC: 0.633) to serology (AUC: 0.614) (p > 0.05).

Conclusion

Histology is the most accurate invasive test, while SAT is the most accurate non-invasive test for *H. pylori* diagnosis in our setting. Despite serology's high sensitivity and low specificity, its predominant use in Cameroon necessitates a pragmatic diagnostic approach. We propose the following algorithm: serology as the first-line assessment, followed by SAT or histology for confirmation in seropositive patients before treatment initiation. This algorithm balances diagnostic accuracy with resource availability and current practice patterns in Cameroon. Larger validation studies are needed to confirm this approach.

## Introduction

*Helicobacter pylori* (*H. pylori*) is a Gram-negative and microaerophilic spiral bacterium that colonizes the gastric mucosa of approximately 50% of the world's population [1]. The prevalence of *H. pylori *infection varies between and within countries due to factors such as age and socioeconomic conditions of the populations. Several epidemiological studies have shown a higher incidence of infection in lower socioeconomic groups and low-income regions [2].

*H. pylori* infection is a major concern today because infection with this bacterium is associated with gastrointestinal diseases such as gastritis, duodenal and gastric ulcers, non-ulcer dyspepsia, gastric adenocarcinoma, and lymphoma [2]. There is thus a need for accurate detection of this organism to facilitate effective treatment and prevention of long-term complications. Currently, treatment of this infection is mainly recommended for patients with ulcers and gastric cancer, or patients who have a high risk of gastric cancer after definite diagnosis of the infection [3].

Both direct and indirect *H. pylori* infection detection methods have been developed. Direct assays that require endoscopy include histopathology, culture, rapid urease test (RUT), and polymerase chain reaction (PCR) [4-6]. Indirect diagnostics include stool antigen tests (SATs), urea breath test (UBT), and serology [7]. Non-invasive diagnostic methods are commonly recommended as first-line tools for the detection of *H. pylori* infection because they are generally less expensive, more convenient for patients, and easier to implement. In addition, these tests can provide an overall assessment of infection status without the need for endoscopic procedures. However, despite these advantages, their diagnostic accuracy may be limited by lower specificity compared with invasive methods, which can result in a higher rate of false-positive results. Consequently, while non-invasive tests are valuable for initial screening, confirmatory testing may be required in certain clinical situations [6].

Stool antigen methods detect *H. pylori* proteins in stool through polyclonal or monoclonal antibodies. However, these test methods show a relatively low sensitivity and specificity of 49% to 92% and 76% to 94%, respectively [8-11]. Moreover, the SAT is preferable for diagnosis in children [12], as its reliability and predictive value in adults are lower [13]. Also, these tests have the ability to distinguish between infected and successfully treated patients [14]. UBT can identify active infection but is affected by comedication and patient compliance, as well as the availability of the test device. Several serological methods have been developed, and the majority are based on enzyme-linked immunosorbent assay formats using purified antigens for IgG antibody detection in plasma or serum [14,15]. They are also cheap and simple to practice, have high sensitivity and high negative predictive values due to cross-reactivity with other antibodies. Moreover, IgG serological methods show lower accuracy due to their inability to differentiate between active and past infection, hence a false-positive result [16-18].

Compared to indirect diagnostics, endoscopy-based methods are still the gold standard, as they permit visualization of the gastric mucosal lining and biopsy sampling for histological analysis or direct culture. Culture methods identify active infection with very high specificity, even if the sensitivity may often vary. However, endoscopy is not always available and, therefore, is not recommended for screening approaches. Moreover, the patchy distribution of *H. pylori* in the gastric mucosa can lead to false-negative results using endoscopy-based tests [19]. 

Although each diagnostic method offers distinct advantages and disadvantages, its performance may vary according to the clinical context [20,21]. The lack of sensitivity will miss patients who have a past infection, while the lack of specificity leads to poor treatment, with consequences such as increased cost, side effects, and the development of resistance.

In Cameroon, UBT is not used for *H. pylori* diagnosis because the equipment is lacking due to its cost. Molecular diagnostic methods, although highly sensitive and specific, are often costly and not widely accessible in many healthcare facilities in Cameroon. Similarly, microbial culture has traditionally been regarded as a reference method for the detection of infectious agents; however, its implementation requires specialized laboratory infrastructure, equipment, and technical expertise that are frequently unavailable in resource-limited settings. Consequently, the routine use of these techniques remains challenging in many developing countries, including Cameroon [22,23]. Consequently, routine diagnosis of *H. pylori* in Cameroon relies on histology, RUT, SAT, and serology, the latter being the most commonly employed method. Given the high prevalence of infection in Cameroon, an inaccurate diagnostic method may lead to poor treatment of uninfected subjects or prevent patients from receiving proper treatment.

In the present study, the choice of PCR targeting the *ureA* gene as a reference standard was based on its high analytical specificity and its ability to detect active *H. pylori* infection. The *ureA* gene encodes the production of urease, an enzyme essential for the successful survival of this bacterium in the acidic gastric environment. It also plays a critical role in colonization and pathogenicity [24,25]. These characteristics make *ureA* a suitable molecular marker for detecting current infection.

Considering that serology is the main test used in our milieu, the current data may bring to light the number of false-positive patients receiving treatment despite not having a current infection, which may help overcome the overestimation of *H. pylori*-related disease risk due to clearance of infection in our milieu.

Our study aims to compare the diagnostic performance of commonly used methods for the detection of *H. pylori* infection and to propose a practical diagnostic algorithm for the diagnosis of *H. pylori* infection in Cameroon and other resource-limited settings.

## Materials and methods

Study design and recruitment of participants

This was a cross-sectional and prospective study conducted at the Gastroenterology Unit of the Bafoussam Regional Hospital, Bafoussam, Cameroon, from October 2022 to May 2023. Participants were recruited consecutively from patients presenting with dyspeptic symptoms who met the study eligibility criteria during the study period. The sample size was determined based on the number of eligible patients available during the study recruitment period, since recruitment was done consecutively. Due to the limited availability of patients undergoing upper gastrointestinal endoscopy at the study site, all consecutive eligible participants were included.

Inclusion criteria

The target population was adult (age ≥ 15 years) patients who had undergone endoscopy for various clinical signs related to gastro-duodenal disorders like dyspepsia. These patients had voluntarily agreed to take part in the study after having signed an informed consent.

Exclusion criteria

Dyspeptic patients who had contraindications to upper endoscopy or colonoscopy, a history of *H. pylori* eradication, recent proton pump inhibitor (PPI) therapy or antibiotic consumption within the last four weeks, frequent use of non-steroidal anti-inflammatory drugs (NSAIDs), or who were non-cooperative were excluded. We also excluded pregnant and breastfeeding women, patients with misidentification, or those who had errors during analysis. This recruitment strategy was adopted to minimize selection bias and better reflect routine clinical practice.

Target condition and reference standard

The target condition investigated in this study was *H. pylori* infection. For each participant, gastric biopsy, blood, and stool samples were collected and analyzed using five diagnostic methods. This included SAT for the detection of *H. pylori *antigens in stool samples, serological testing for the detection of *H. pylori*-specific IgG antibodies in blood samples, and three biopsy-based methods: RUT, histopathological examination, and PCR. The index tests evaluated were those commonly used for the diagnosis of *H. pylori* infection in our milieu, namely serology, SAT, RUT, and histology. The diagnostic performance of each index test was assessed by comparing its results with those obtained using PCR, which served as the reference standard. A positive diagnosis of *H. pylori *infection was defined by the detection of the *ureA* gene in DNA extracted from gastric biopsy specimens using PCR.

Data collection

A structured questionnaire was used to collect the following information from the patients (Appendix 1): sociodemographic data (age and sex), information related to lifestyle (income level, smoking, and alcohol consumption), personal medication history (history of drug consumption, such as antibiotics, PPIs, bismuth salts, and NSAIDs), as well as family history of gastric cancer.

Samples collection

Endoscopy (EGD) and Biopsy Collection

Gastric biopsies were collected by an experienced resident gastroenterologist. The volunteers fasted for at least eight hours before the procedure. The procedure was carried out after pharyngeal anesthesia with lidocaine. To avoid contamination, the endoscope was carefully cleaned and disinfected before and after each procedure. Biopsy specimens were collected from the greater curvature of the antrum, from the upper body of the stomach, or from the gastric angulus or other locations, if required. The ﬁrst biopsy specimen was inoculated immediately into a screw-capped bottle containing urea media for RUT. The next specimen was ﬁxed in 10% buﬀered formalin for histological examination. Another biopsy specimen was collected in brain infusion medium (BHI) for PCR and stored at -80°C until DNA extraction was performed.

Blood Sample Collection

Three milliliters (3 mL) of venous blood were collected from each participant and transferred into a clean, dry test tube. The blood was allowed to clot at room temperature for approximately 10 minutes. Serum was subsequently separated by centrifugation at 3000 rpm for five minutes. The resulting serum samples were then used for the detection of *H. pylori*-specific antibodies using the *H. pylori* Antibody Rapid Test (Quidao Hightop Biotech Co., Ltd. China).

Stool Sample Collection

Each participant was asked to provide a stool sample, which was collected in a sterile stool container. The specimens were subsequently analyzed for the presence of *H. pylori* antigens using the immunochromatographic *H. pylori* Antigen Rapid Test Cassette (Healgen Scientific Limited Liability Company, Houston, TX, USA) according to the manufacturer's instructions.

Sample Analysis

To reduce bias, laboratory analyses were blinded, and histological slides were evaluated by a pathologist who was blinded to the results of PCR, RUT, serology, and SAT.

Rapid Urease Test (RUT)

RUT was carried out using urea media (Biolab Diagnostics Laboratory Inc., Budapest, Hungary). Prior to the test, the medium was prepared according to the manufacturer's instructions. Biopsy specimens, immediately after collection, were inoculated into a screw-capped bottle containing urea media. A positive result was indicated by a change in color from yellow to pink within 24 hours after incubation at 37°C.

Serology Test

*H. pylori* Antibody Rapid Test Kit was used to qualitatively detect *H. pylori* IgG antibodies in serum. The test is a double-antigen sandwich method used to detect *H. pylori* antibodies in serum or plasma. Summarily, one drop (25 µL) of serum was added to the sample well of the cassette, and two drops of sample diluent provided by the kit were added. The result was read within 15-20 minutes. The presence of two distinct colored lines was indicative of a positive result, whereas only one colored line indicated a negative result.

Stool Antigen Test (SAT)

Detection of *H. pylori* in stool samples was performed using the *H. pylori* Ag Rapid Test Cassette Kit. The cassette was labeled with the participant identification number. Then, 50 mg of stool was collected with a specimen applicator and applied to a specimen collection tube containing dilution buffer. The specimen tube was then shaken vigorously to mix the specimen and buffer and was allowed to stand for two minutes. Later, the tip of the specimen collection tube was broken off to squeeze two drops of sample solution into the sample well of the cassette. The results were read after 10-15 minutes. Positive samples demonstrated two red lines, whereas negative samples had only one red line on the test strip.

Histological Examination

The biopsy samples fixed in 10% formalin were later removed from formalin and inserted into an accessioned cassette. The cassette was placed into a tissue-processing automator for dehydration and clearing. The biopsy obtained at the end of this tissue-processing step was embedded in paraffin and cut into fine (4 μm) sections using a microtome. Four-micron-thick histological sections of each fragment were obtained and mounted on glass slides. The slides were stained using Giemsa stain and examined by a histopathologist to detect the presence or absence of *H. pylori*.

PCR Detection

Biopsy samples for PCR analysis were collected in BHI and stored at -80°C until DNA extraction. DNA extraction was performed using the phenol-chloroform method with a phenol-chloroform-isoamyl alcohol mixture (Sigma-Aldrich, Merck KGaA, Darmstadt, Germany).

The presence of *H. pylori* was confirmed by PCR amplification targeting the specific *H. pylori* *ureA* gene, corresponding to an amplicon of 411 bp, with the primer set as follows: forward 5′-GCCAATGGTAAATTAGTT-3′ and reverse 5′-CTCCTTAATTGTTTTTAC-3′ [26].

PCR was carried out in a 25 μL reaction mixture containing 12.5 μL of Master Mix Ready-to-Use CoTapR Green Master (Promega Corporation, Madison, WI, USA), Taq DNA polymerase, dNTPs, and MgCl₂, 4 μL of DNA template, 2.5 μL of forward (F) primer, 2.5 μL of reverse (R) primer, and 3.5 μL of nuclease-free water. Samples were run in duplicate and were considered positive if both reactions were positive. For each batch of PCR assays, nuclease-free water was used as the negative control, and the *H. pylori* reference strain PMSS1 was used as the positive control.

PCR conditions were as follows: denaturation at 95°C for three minutes, followed by 35 cycles at 95°C for 30 seconds, 56°C for 30 seconds, and 72°C for one minute, with a final extension at 72°C for five minutes in a thermocycler (Bio-Rad Laboratories, Inc., Hercules, CA, USA).

After amplification, the PCR products (5 μL) were verified using 1.5% agarose gels stained with ethidium bromide (0.1 μL/mL; RotiR-GelStain, Carl Roth GmbH, Karlsruhe, Germany) and photographed under UV light. A 1 Kb Plus DNA ladder (5-10 μL) purchased from Thermo Scientific (Thermo Fisher Scientific Inc., Waltham, MA, USA), GeneRuler, was used as a size marker.

Algorithm Development

The proposed diagnostic algorithm was derived from the comparative performance of the evaluated diagnostic methods and their practical applicability in the Cameroonian healthcare setting. Test combinations were assessed using their observed sensitivity, specificity, predictive values, area under the curve (AUC) values, and overall diagnostic accuracy. Priority was given to diagnostic methods that demonstrated acceptable diagnostic performance, affordability, and availability in resource-limited healthcare facilities. The algorithm was derived from the findings of the present study and existing evidence and was intended as a practical guide rather than a validated clinical decision tool.

Statistical analysis

Statistical analysis was performed using RStudio version 4.4.1 (RStudio, Inc., Boston, MA, USA). Continuous variables were expressed as the mean ± standard deviation (SD), and comparisons between age groups were performed using Student's t-test. Categorical variables were expressed as frequencies and percentages. Concordance between diagnostic methods was expressed as the percentage agreement, and differences in paired *H. pylori* positivity rates were evaluated using the Chi-square test with corresponding p-values. Sensitivity, specificity, positive predictive value (PPV), negative predictive value (NPV), and accuracy of the histology test, RUT, SAT, and serology test were calculated as universally defined, using PCR as the reference standard.

Receiver operating characteristic (ROC) curve analysis was performed to evaluate test performance, and the corresponding AUC with 95% confidence intervals was calculated. Pairwise comparisons of correlated ROC curves were performed using DeLong's test, with statistical significance determined using the corresponding p-values. A p-value < 0.05 was considered statistically significant.

## Results

Characteristics of the study population and *H. pylori* infection

This study included 82 consecutive dyspeptic subjects attending the Gastroenterology Unit of the selected health facility. Of the 82 subjects, 49 (59.75%) were women, and 33 (40.24%) were men. The age group of 55 to 64 years was the most represented (19; 23.5%). The prevalence of *H. pylori* infection was 79.26% based on the PCR diagnostic method as the gold standard. The most affected age groups were 55-64 years (23.0%, 15/65) and 45-54 years (21.5%, 14/65), followed by the age groups 35-44 years (15.4%, 10/65) and >65 years (16.9%, 11/65). This difference was not significant (χ² = 1.741, p = 0.88). The mean age among infected patients was 49.25 ± 17.68 years versus 48.29 ± 15.32 years among uninfected patients, but the difference was not significant (t = 0.203, p = 0.84). Regarding gender, 36.9% of males versus 63.1% of females were found to be infected. This difference was not significant (χ² = 1.49, p = 0.47) (Table 1).

**Table 1:** Distribution of H. pylori status based on PCR test according to demographic characteristics N: number (percentage); X^2^: Chi-square; t: student test; +ve: positive; -ve: negative; SD: standard deviation; *H. pylori*: *Helicobacter pylori*; PCR: polymerase chain reaction

Variables	Total number (%), N = 82	*H. pylori* +ve, N = 65 (79.26%)	*H. pylori* -ve, N = 17 (20.74)	X^2^ (p-value)
Gender				
Male	33 (40.24)	24 (36.9)	9 (52.9)	1.49 (0.47)
Female	49 (59.75)	41 (63.1)	8 (47.1)	
Age (years), mean age ± SD	48.77 ± 16.50	49.25 ± 17.68	48.29 ± 15.32	t = 0.203, p = 0.84
≤ 24	10 (12.1)	9 (13.85)	1 (5.9)	1.741 (0.88)
25-34	9 (10.9)	6 (9.2)	3 (17.6)	
35-44	13 (15.8)	10 (15.3)	3 (17.6)	
45-54	18 (21.9)	14 (21.5)	4 (23.5)	
55-64	19 (23.1)	15 (23.0)	4 (23.5)	
> 65	13 (15.8)	11 (16.9)	2 (11.8)	

*H. pylori* in the study population based on PCR detection

**Figure 1:**
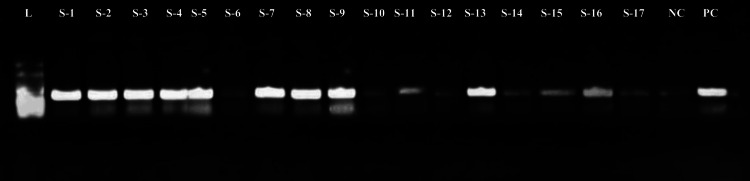
PCR gel electrophoresis of the H. pylori ureA gene (411bp) Lane L: ladder; PC: positive control; NC: negative control; Lanes S1-S17: representative patient biopsy samples; *H. pylori*:* Helicobacter pylori*; PCR: polymerase chain reaction

The results of electrophoresis for the *ureA* gene PCR amplicons from gastric biopsy DNA are shown in Figure 1.

Comparison of *H. pylori* positivity between diagnostic methods

*H. pylori* infection was detected in 62.2% (51/82), 45.1% (37/82), 76.8% (63/82), and 73.2% (60/82) based on SAT, histology, serology, and RUT, respectively.

PCR detected the highest proportion of *H. pylori*-positive cases (79.3%), followed by serology (76.8%), RUT (73.2%), SAT (62.2%), and histology (45.1%). Significant differences in positivity rates were observed between PCR and histology (p < 0.0001), histology and RUT (p = 0.0003), PCR and SAT (P = 0.016), histology and SAT (p = 0.028), and histology and serology (p < 0.0001). In contrast, positivity rates did not differ significantly between PCR and RUT, PCR and serology, RUT and SAT, or RUT and serology (Table 2).

**Table 2:** Comparison of H. pylori positivity between diagnostic methods Differences between diagnostic methods were evaluated using the Chi-square test. Statistical significance was defined as p < 0.05. “*” denotes statistically significant p-values. SAT: stool antigen test; RUT: rapid urease test; PCR: polymerase chain reaction

Diagnostic test	Positivity rate (%)	X^2^	p-value
PCR vs Histology	79.26 vs 45.1	20.33	< 0.0001*
PCR vs RUT	79.26 vs 73.2	0.8410	0.3591
Histology vs RUT	45.1 vs 73.2	13.35	0.0003*
PCR vs Serology	79.26 vs 76.8	0.1424	0.7059
PCR vs SAT	79.26 vs 62.2	5.773	0.0163*
Histology vs SAT	45.1 vs 62.2	4.806	0.0284*
Histology vs Serology	45.1 vs 76.8	17.32	< 0.0001*
RUT vs SAT	73.2 vs 62.2	2.258	0.1329
RUT vs Serology	73.2 vs 76.8	0.2927	0.5885

Comparison of *H. pylori* positivity agreement between diagnostic methods

Agreement between methods ranged from 51.22% to 73.17%, with the highest agreement observed between PCR and serology, while the lowest was observed between histology and serology. Significant differences in agreements were observed between PCR and histology (58.54%; p = 0.0013), histology and RUT (57.32%; p = 0.0001), PCR and SAT (65.85%; p = 0.0401), RUT and SAT (59.76%; p = 0.0019), and RUT and serology (64.63%; p = 0.0021). These findings demonstrate that the estimated prevalence of *H. pylori* infection varies substantially according to the diagnostic method employed (Table 3).

**Table 3:** Comparison of H. pylori positivity agreement between diagnostic methods Agreement represents the proportion of concordant results between the compared diagnostic methods. Statistical significance was defined as p < 0.05. “*” denotes statistically significant p-values. SAT: stool antigen test; RUT: rapid urease test; PCR: polymerase chain reaction; *H. pylori*: *Helicobacter pylori*

Diagnostic test	Agreement (%)	p-value
PCR vs Histology	58.54	0.0013*
PCR vs RUT	57.32	0.7939
Histology vs RUT	57.32	0.0001*
PCR vs Serology	73.17	0.050
PCR vs SAT	65.85	0.0401*
Histology vs SAT	60.98	0.602
Histology vs Serology	51.22	0.48
RUT vs SAT	59.76	0.0019*
RUT vs Serology	64.63	0.0021*

Diagnostic performance between invasive methods for *H. pylori* detection

The diagnostic performance of different invasive tests used for *H. pylori* detection was evaluated.

PCR Testing Versus Histology

Taking PCR as the reference standard, the sensitivity and specificity of histology were 52.31% and 82.35%, respectively. The PPV and NPV were 91.89% and 31.11%, respectively.

PCR Testing Versus RUT

Taking PCR as the reference standard, the sensitivity and specificity of RUT were 76.92% and 41.18%, respectively. The PPV and NPV were 83.33% and 31.81%, respectively.

Histology Versus RUT

Taking RUT as the reference standard, the sensitivity of histology was 51.67%, and its specificity was 72.73%. The PPV and NPV were 83.76% and 35.56%, respectively. The accuracy of the histology test was 57.32% (Table 4).

**Table 4:** Comparison of invasive methods in the diagnosis of H. pylori infection Diagnostic performance metrics were calculated: sensitivity, specificity, predictive values, and accuracy are expressed as percentages. SAT: stool antigen test; RUT: rapid urease test; PCR: polymerase chain reaction; TP: true positive; FP: false positive; TN: true negative; FN: False negative; PPV: positive predictive value; NPV: negative predictive value; *H. pylori*: *Helicobacter pylori*

Diagnostic test	Reference standard (PCR)			Sensitivity (%)	Specificity (%)	PPV (%)	NPV (%)	Accuracy (%)
	Positive	Negative	Total					
	65	17	82					
Histology				52.31	82.35	91.89	31.11	58.54
Positive	34 (TP)	3 (FP)	37					
Negative	31 (FN)	14 (TN)	45					
RUT				76.92	41.18	83.33	31.81	57.32
Positive	50 (TP)	10 (FP)	60					
Negative	15 (FN)	7 (TN)	22					
	Reference standard (RUT)							
	60	22	82					
Histology				51.67	72.73	83.76	35.56	57.32
Positive	31 (TP)	29 (FP)	60					
Negative	6 (FN)	16 (TN)	22					

Discriminatory power of histology versus RUT based on PCR results using the AUC value

**Figure 2:**
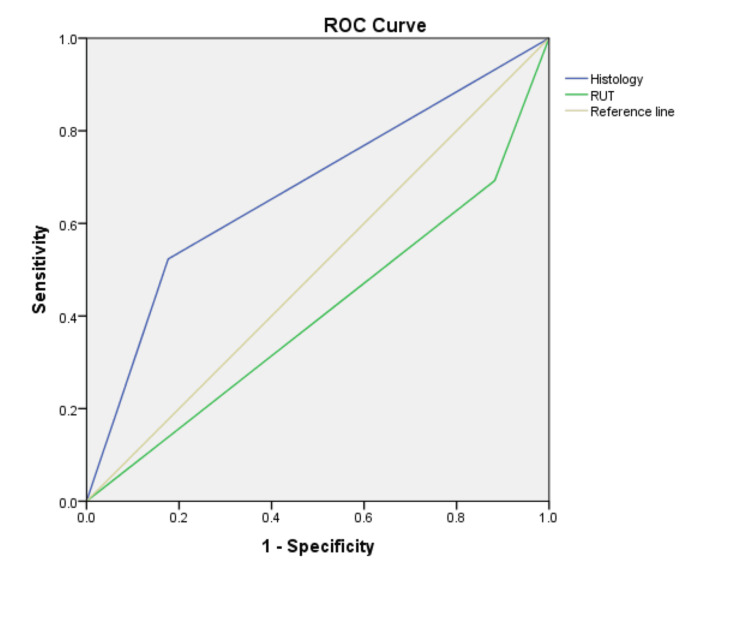
ROC curves comparing histology and rapid urease test using PCR as the reference standard The diagonal reference line represents a test with no discriminatory capacity (AUC = 0.50). Histology had an AUC of 0.673 (95% CI: 0.538-0.808), while RUT had an AUC of 0.405 (95% CI: 0.263-0.547). ROC: receiver operating characteristic; PCR: polymerase chain reaction; RUT: rapid urease test; AUC: area under the curve

ROC curve analysis demonstrated that histology had an AUC of 0.673 (95% CI: 0.538-0.808), which was significantly greater than 0.50 (p = 0.02), indicating fair discriminatory ability. In contrast, the RUT had an AUC of 0.405 (95% CI: 0.263-0.547), which was not significantly different from 0.50 (p = 0.23), indicating poor discriminatory performance. Pairwise comparison of the correlated ROC curves using DeLong’s test demonstrated that the AUC of histology was significantly higher than that of the RUT (p < 0.001), indicating superior discriminatory performance of histology in this study. The corresponding ROC curve is presented in Figure 2.

Comparison of non-invasive methods for *H. pylori* detection using invasive methods as the reference standard

The diagnostic performance of different non-invasive tests used for *H. pylori* detection was evaluated.

PCR Testing Versus Serology

Taking PCR as the reference standard, the sensitivity of serology was 81.54%, and its specificity was 41.18%. The PPV and NPV were 84.13% and 36.84%, respectively. The accuracy of the serology test was 73.17%.

PCR Testing Versus SAT

Taking PCR as the reference standard, the sensitivity of SAT was 67.69%, and its specificity was 58.82%. The PPV and NPV were 86.27% and 32.26%, respectively.

Histology Testing Versus SAT

Taking histology as the reference standard, the sensitivity of SAT was 75.68%, and its specificity was 48.89%. The PPV and NPV were 54.90% and 70.97%, respectively. The accuracy of SAT was 60.98%.

Histology Testing Versus Serology

Taking histology as the reference standard, the sensitivity of serology was 81.08%, and its specificity was 26.67%. The PPV and NPV were 47.62% and 63.16%, respectively. The accuracy of serology was 51.22%.

RUT Testing Versus SAT

Taking RUT as the reference standard, the sensitivity of SAT was 65%, and its specificity was 45.45%. The PPV and NPV were 32.26% and 76.47%, respectively. The accuracy of the SAT was 59.76%.

RUT Testing Versus Serology

Taking RUT as the reference standard, the sensitivity of serology was 75.6%, and its specificity was 31.58%. The PPV and NPV were 78.33% and 27.27%, respectively. The accuracy of RUT was 64.63% (Table 5).

**Table 5:** Comparison of serology and SAT based on invasive methods as reference standard Diagnostic performance metrics were calculated. Sensitivity, specificity, predictive values, and accuracy are expressed as percentages. SAT: stool antigen test; RUT: rapid urease test; PCR: polymerase chain reaction; PPV: positive predictive value; NPV: negative predictive value

Diagnostic test	Reference standard (histology)			Sensitivity (%)	Specificity (%)	PPV (%)	NPV (%)	Accuracy (%)
	Positive	Negative	Total					
	37	45	82					
SAT				75.68	48.89	54.90	70.97	60.98
Positive	28 (TP)	23 (FP)	51					
Negative	9 (FN)	22 (TN)	31					
Serology				81.08	26.67	47.62	63.16	51.22
Positive	30 (TP)	33 (FP)	63					
Negative	7 (FN)	12 (TN)	19					
	Reference standard (RUT)							
	60	22						
SAT								
Positive	39 (TP)	12 (FP)	51	65	45.45	76.47	32.26	59.76
Negative	21 (FN)	10 (TN)	31					
Serology				78.33	27.27	74.60	31.58	64.63
Positive	47 (TP)	16 (FP)	63					
Negative	13 (FN)	6 (TN)	19					
	Reference standard (PCR)							
	65	17	82					
SAT								
Positive	44 (TP)	7 (FP)	51	67.69	58.82	86.27	32.26	65.85
Negative	21 (FN)	10 (TN)	31					
Serology								
Positive	53 (TP)	10 (FP)	63	81.54	41.18	84.13	36.84	73.17
Negative	12 (FN)	7 (TN)	19					

Discriminatory power of serology versus SAT using the AUC value

Serology Versus SAT Using PCR as Reference Standard

**Figure 3:**
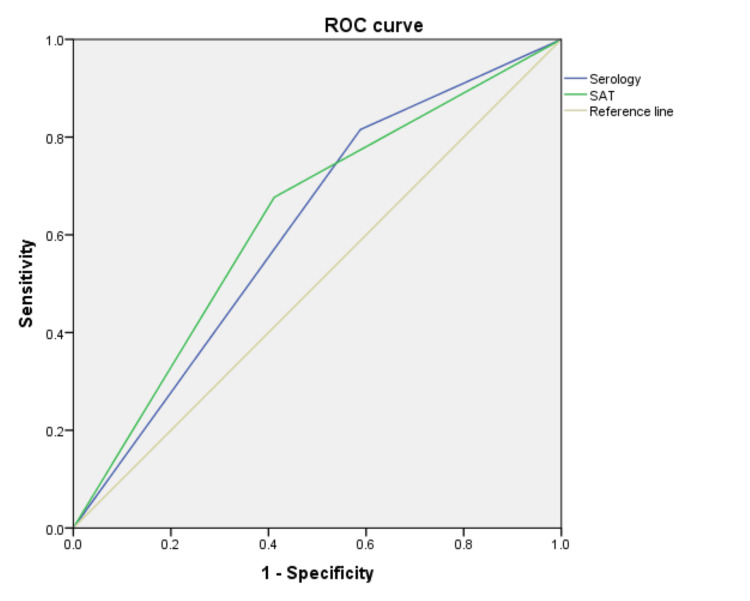
ROC curves comparing serology and SAT using PCR as the reference standard The diagonal reference line represents a test with no discriminatory capacity (AUC = 0.50). Serolgy AUC of 0.614 (95% CI: 0.538-0.808) while SAT AUC 0.633 (95% CI: 0.481-0.784). ROC: receiver operating characteristic; PCR: polymerase chain reaction; RUT: rapid urease test; AUC: area under the curve; SAT: stool antigen testing

The ROC curve analysis demonstrated that serology had an AUC of 0.614 (95% CI: 0.538-0.808), while SAT had an AUC of 0.633 (95% CI: 0.481-0.784). Although SAT showed a slightly higher AUC than serology, neither AUC was significantly different from 0.50. Also, pairwise comparison of the correlated ROC curves using DeLong's test showed no statistically significant difference between the two AUCs (p > 0.05). This implies that both diagnostic methods have similar fair discriminatory ability. The corresponding ROC curve is presented in Figure 3.

Serology Versus SAT Using Histology as Reference Standard

**Figure 4:**
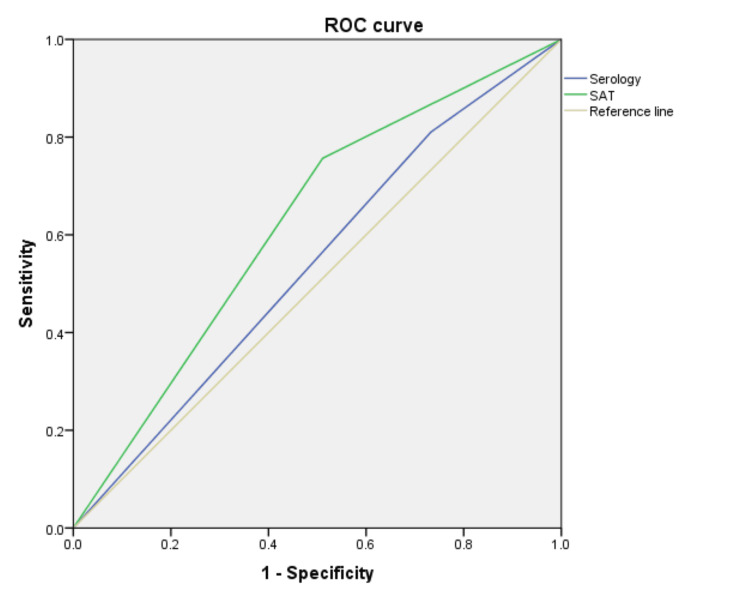
ROC curves comparing serology and SAT using histology as the reference standard The diagonal reference line represents a test with no discriminatory capacity (AUC = 0.50). Serology AUC of 0.539 (95% CI: 0.413-0.664), while SAT AUC of 0.623 (95% CI: 0.501-0.770). ROC: receiver operating characteristic; AUC: area under the curve; SAT: stool antigen testing

ROC curve analysis showed that serology had an AUC of 0.539 (95% CI: 0.413-0.664) at p = 0.548, while SAT had an AUC of 0.623 (95% CI: 0.501-0.770) at p = 0.057. Although SAT showed a numerically higher AUC than serology, neither AUC differed significantly from 0.50. Comparison of the correlated ROC curves using DeLong’s test also showed no statistically significant difference between the two AUCs (p > 0.05), indicating comparable discriminatory performance between serology and SAT relative to histology as the reference standard. The corresponding ROC curve is presented in Figure 4.

Serology Versus SAT Using RUT as Reference Standard

**Figure 5:**
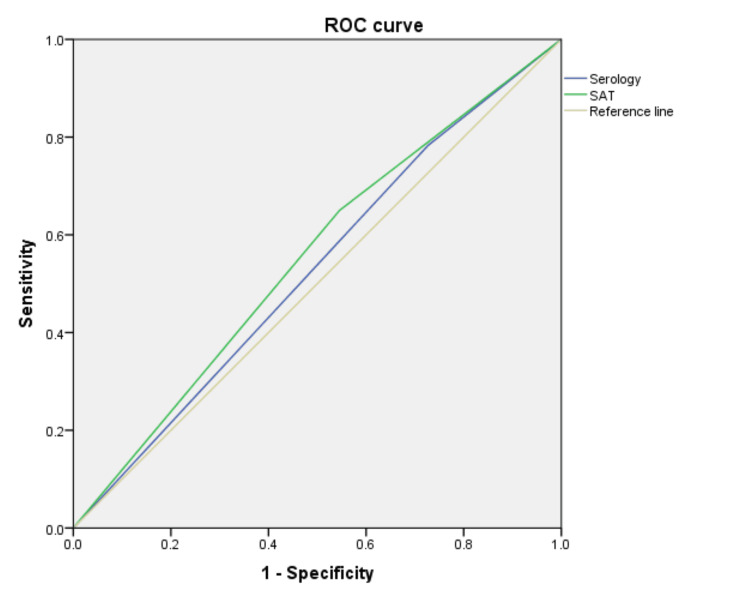
ROC curves comparing serology and SAT using RUT as the reference standard The diagonal reference line represents a test with no discriminatory capacity (AUC = 0.50). Serology AUC of 0.528 (95% CI: 0.385-0.672), while SAT AUC was 0.525 (95% CI: 0.410-0.695). ROC: receiver operating characteristic; RUT: rapid urease test; AUC: area under the curve; SAT: stool antigen testing

The ROC curve analysis demonstrated that serology had an AUC of 0.528 (95% CI: 0.385-0.672), while SAT had an AUC of 0.525 (95% CI: 0.410-0.695). Neither AUC was significantly different from 0.50 (both p > 0.05). Furthermore, comparison of the two correlated ROC curves using DeLong's test showed no statistically significant difference between the AUCs (p > 0.05), indicating comparable discriminatory performance between serology and SAT. The corresponding ROC curve is presented in Figure 5.

## Discussion

The selection of an appropriate diagnostic test should be guided by the clinical context, the diagnostic performance of the available methods, and the cost-effectiveness of the overall testing strategy [27]. While a lack of sensitivity will miss patients who should be treated, a lack of specificity leads to unnecessary treatments with consequences such as cost, side effects, and aggravation of cross-resistance.

In this study, *H. pylori* infection varied according to diagnostic methods, with prevalence rates of 62.2% by SAT, 73.2% by RUT, 45.1% by histology, 76.8% by serology, and 79.3% by PCR. These findings show that the infection status of an individual depends on the diagnostic method used and underline the need for reliable, available, and affordable diagnostic methods to facilitate effective treatment and prevention of long-term complications.

The current prevalence of 62.2% obtained with SAT is comparable to the 59.0% reported by Sai Priya et al. [28] but higher than the 45% previously reported in the Littoral Region of Cameroon [11], the 47.4% reported at Melong District Hospital of Cameroon [29], the 52.27% reported among asymptomatic children in Buea and Limbe Health Districts of Cameroon [30], and the 26.82% rate reported in Israel [31]. These differences in prevalence reflect variations in geographical location, sanitation, socioeconomic conditions, study period, and study population. The prevalence of *H. pylori* infection using SAT was significantly higher among women than men. A similar observation was reported in the city of Bamenda, Cameroon [32], whereas several studies reported no significant association between sex and this infection [33-35]. In contrast, a previous study showed that more males were positive than females [36]. This can be due to the greater proportion of females in our sample population or to the fact that more women seek consultations.

Using the RUT method, the prevalence of *H. pylori* infection was 73.2%, closely similar to the 72.5% reported among symptomatic patients in Yaoundé, Cameroon [37]. This similarity in prevalence may explain the high incidence of *H. pylori* infection in our setting. However, this estimate was higher than the prevalence reported using RUT among dyspeptic patients in the Littoral Region of Cameroon (43%) [11] and among dyspeptic outpatients in reference hospitals in the West and Centre Regions of Cameroon (57.9%) [36]. Such observations suggest a change in the trend of *H. pylori* infection over time in our setting. In contrast, higher prevalences have been reported in other settings [38,39]. These variations likely reflect differences in population characteristics and disease severity.

Histological examination identified *H. pylori* in 45.1% of participants which is lower than the 65.94% reported in reference hospitals in the West and Centre Regions in Cameroon [40] and 75.58% reported by Chowdhury et al. [38], but comparable to the 48.35% reported previously by Khalifehgholi et al. [41] Differences in biopsy sampling, bacterial load distribution within the gastric mucosa, tissue processing, and the expertise of the pathologist may explain these discrepancies.

The prevalence of the infection based on the serological test was 76.8%, which was higher than the 64.39% rate reported in the Littoral Region of Cameroon [36], the 43.4% reported in the Melong West Region of Cameroon [29], and the 27.5% reported in Bamenda, Cameroon [32]. These discrepancies could be due to the fact that serological tests detect antibodies that may persist after successful eradication and, as such, cannot reliably distinguish active from previous infection. Consequently, serology tends to produce higher positivity rates than tests that directly detect the organism.

The prevalence of the infection based on the PCR method was 79.3%, which is higher than the 34.01% reported in a descriptive cross-sectional study by Nkoth et al. in Yaoundé, Cameroon, using a molecular test [42]. This difference can be explained by the fact that both molecular methods targeted different genes of *H. pylori*. It could also be due to the detection limit of the primers used.

Accuracy, sensitivity, and specificity among tests evaluated as diagnostic performance

For invasive tests, using PCR as the reference standard, histology and RUT showed overall accuracies of 58.54% and 57.32%, respectively. Histology demonstrated relatively high specificity (82.35%) but low sensitivity (52.31%), whereas RUT showed higher sensitivity (76.92%) but lower specificity (41.18%). These findings show that histology was more effective in identifying uninfected individuals, while RUT was more sensitive for detecting infected patients, although both exhibited moderate overall diagnostic performance. The diagnostic accuracy of invasive tests was consistent with previous studies demonstrating the performance of biopsy-based methods [43,44]. However, the present accuracy of RUT was lower than the 69.7% reported by Kismat et al. [45]. This discrepancy may be due to bacterial density in the biopsy specimen, the site of collection, and the size of biopsy samples [46]. In addition, the patchy distribution of *H. pylori* in gastric mucosa can lead to false-negative results, thereby reducing the specificity and accuracy of the test. Also, RUT may produce false-positive reactions because of other urease-producing microorganisms colonizing the stomach. Histology showed a higher specificity than that reported by Abideen et al. [47], which could be useful in confirming *H. pylori* infection. Nevertheless, histology presents limitations, including higher cost, long formalin processing time, and inter-observer variation in assessment, which could reduce its applicability in routine clinical practice. In fact, differences in visual observation by histopathologists in clinical settings and occasional errors in staining procedures, such as contamination with formalin during long-term processing, may lead to false-negative results [48].

When comparing histology and PCR testing, we found significant differences in *H. pylori *positivity (p < 0.0001) and discordance of results (p = 0.0013) between the two tests, indicating that both tests have substantial discrepancies and suggesting that histological examination alone may underestimate active infection in some individuals. The blinded assessment of biopsy specimens by pathologists in this study helped minimize observer bias, although it could not eliminate limitations related to sampling variability and bacterial distribution. PCR detection may produce false-positive results due to genetic similarity with non-*H. *pylori species and false-negative results due to low bacterial load and the presence of PCR inhibitors [49]. Our blinded review by pathologists minimized bias. Moreover, the presence of two forms of *H. pylori* (spiral and coccoid forms) could explain the false-negative results in histology [49].

When compared with PCR as the reference standard, histology demonstrated a significantly greater ability to discriminate between infected and uninfected individuals than RUT (p < 0.001) based on ROC curve AUC analysis. The superior performance of histology may be attributable to its higher specificity, although its sensitivity remained limited because of factors such as the patchy distribution of *H. pylori* within the gastric mucosa and biopsy sampling variability.

For non-invasive tests, using PCR as the reference standard, serology and SAT revealed overall accuracies of 73.17% and 65.85%, respectively. Serology demonstrated higher sensitivity (81.54%) but lower specificity (41.18%), whereas SAT showed lower sensitivity (67.69%) but higher specificity (58.82%). These findings may suggest that serology is more suitable for identifying infected individuals, while SAT provides better discrimination of uninfected individuals. The accuracy of serology observed in this study is consistent with previous reports of 86% by Iqbal et al. [50] and 61% by Kouitcheu Mabeku et al. [11]. Similarly, the current sensitivity of serology was comparable to the 95% sensitivity found by Iqbal et al. [50].

However, the specificity of serology observed in our study was lower than the 80% reported by the same authors [50] but was similar to the 39.65% reported by Kouitcheu Mabeku et al. [11]. In contrast, a lower sensitivity of 70% and a remarkably lower specificity of 12.5% have been reported [47]. These differences highlight the variability in the diagnostic performance of serological assays across different settings. This could be explained by differences in study populations, the diagnostic kits used, or persistent circulating antibodies, which make it difficult to distinguish between active and previous infection.

The accuracy of SAT observed in this study was comparable to the 67.79% previously reported [4], whereas Abideen et al. reported a lower accuracy of 47.1% [47]. Likewise, the sensitivity and specificity of SAT in our study were comparable to the 77.79% and 59.37%, respectively, reported by AbuTaleb et al. [7]. In contrast, considerably higher sensitivity (96.3%) and specificity (91.66%) have been reported elsewhere [51]. These variations could be due to differences in bacterial load, stool sample handling and storage, or the commercial test kits used. Despite its moderate sensitivity and specificity in our study, SAT remains a practical non-invasive method for detecting active *H. pylori* infection because it identifies bacterial antigens rather than antibodies, making it more suitable than serology for confirming current infection.

This study demonstrated significant differences in *H. pylori* positivity (p = 0.0163) and discordance of results (p = 0.0401) between the SAT and PCR tests. In addition, SAT and histology showed significantly different positivity rates (p = 0.0284), further highlighting the diagnostic variability. The performance of SAT depends on the concentration of *H. pylori* antigens excreted in the stool, leading to low levels of fecal antigen and false-negative results [52]. Conversely, the diagnostic performance of histology depends on several factors, such as the number of biopsy specimens, the density and distribution of *H. pylori* within the gastric mucosa, and tissue processing [53]. Together, these factors may explain the observed discordance between SAT, histology, and PCR in the present study.

When compared with PCR as the reference standard, both SAT and serology demonstrated non-significant modest diagnostic discrimination (p > 0.05) based on the ROC AUC analysis. Although SAT exhibited a slightly higher AUC than serology, our result is similar to previous studies revealing SAT as a reliable, simple-to-perform, and painless test for the detection of *H. pylori* infection [11,54]. Previous studies have reported SAT as a suitable non-invasive method for clinical practice, treatment, or monitoring post-treatment of this infection [46]. Although serology and SATs have their own preferences and drawbacks [21], cross-reactivity between antibodies against closely related *H. pylori* microorganisms and *H. pylori* antigens may lead to a decrease in the discriminatory power of SAT. However, positive cases reported by serology may not differentiate active from past infection and cannot be used to indicate eradication therapy for *H. pylori* because antibodies may persist even after eradication of the bacteria [55].

Clinicians practicing in resource-limited settings should consider serology as an initial screening tool when SAT is unavailable. Whenever feasible, positive serological results should be confirmed using SAT before treatment decisions are made. Thus, histology can be used as a confirmatory test in our milieu, where facilities are present. Regarding non-invasive tests, SAT can be considered the best and most reliable non-invasive test due to its high specificity and its ability to distinguish between positive and negative subjects. Thus, SAT can be used for current infection diagnosis and follow-up in our milieu.

Limitations

Despite the encouraging findings, several limitations should be acknowledged. The relatively small sample size and the limited number of PCR-negative cases may have reduced the statistical power and affected the precision of some diagnostic performance estimates. In addition, participants were recruited from selected healthcare facilities, which may limit the generalizability of the findings to the broader Cameroonian population. Finally, larger multicenter studies with a greater number of samples are needed to validate its diagnostic performance and confirm its applicability in routine clinical practice, particularly in resource-limited settings.

## Conclusions

The prevalence of *H. pylori *infection varied according to the diagnostic method used, highlighting the importance of test selection in clinical practice. Among invasive methods, histology demonstrated greater specificity and discriminatory ability than RUT, supporting its role as a confirmatory test when endoscopic facilities are available. Among non-invasive methods, SAT showed better specificity and diagnostic performance for active infection, whereas serology exhibited higher sensitivity and may be useful for initial screening. We propose an algorithm for diagnosing *H. pylori* consisting of serology as the first-line assessment of *H. pylori* infection, followed by SAT or histology for confirmation before any treatment administration in our resource-limited settings or milieu.

## Appendices

Appendix 1

I. Participant identification

Date: …………………………………………………….

1. Code…………………………………….…………………………………...

2. Age…........... 

3. Gender ………………….

4. Civil status ……………………

5. Region of residence ………………………….

6. Town of residence ……………………

7. Level of education ……………………

II. Risk factors for *Helicobacter pylori*

8. Profession: Health personnel ☐; Others ☐; Please precise: ………………….

9. Socio-economic level: Low ☐; Middle ☐; High ☐

10. What was your childhood area of residence? Rural area ☐; Urban area ☐

11. How many people are there per sleeping room? Less than 4 ☐; Greater than 4 ☐; Exactly 4 ☐

12. What type of water do you usually consume? Well ☐; Tap ☐; Underground ☐; Other ☐; Please precise: …………………

13. Do you usually consume spiced food? Yes ☐; Sometimes ☐; No ☐

14. Smoking status: Yes ☐; No ☐;​​​​​​​ If no, do you live with a smoker? ☐

15. Alcohol consumer? Yes ☐; No ☐

16. Have you been under medication these past six weeks? Yes ☐; No ☐; If yes, please precise: ……………………………………………………​​​​​​​

17. Has someone in your family suffered from gastric cancer? Yes ☐; No ☐

III. Clinical signs and symptoms of *Helicobacter pylori*

18. Do you experience epigastric burns? Yes ☐; Sometimes ☐; No ☐

19. Do you experience epigastric pains? Yes ☐; No ☐

20. Do you have nausea? Yes ☐; Sometimes ☐; No ☐

21. Do you vomit frequently? Yes ☐; Sometime ☐; Non ☐

22. Do you experience abdominal discomfort such as flatulence and bloating? Ressentez-vous des inconforts abdominaux à type de flatulence et de ballonnement?​​​​​​​ Yes ☐; Sometimes ☐; No ☐

Bias Factors

23. Please tick the drugs you’ve consumed these past six weeks 

Amoxicilline ☐; Tétracycline ☐; Clarithromycine ☐; Metronidazole ☐;​​​​​​​Levofloxacine ☐; None ☐

24. Do you generally consume antacids or AINS for gastric pain? Yes ☐; No ☐; If yes, which? …………………..

Thank you for your participation
